# Aptamer-Conjugated Tb(III)-Doped Silica Nanoparticles for Luminescent Detection of Leukemia Cells

**DOI:** 10.3390/biomedicines8010014

**Published:** 2020-01-13

**Authors:** Yaroslav A. Grechkin, Svetlana L. Grechkina, Emil A. Zaripov, Svetlana V. Fedorenko, Asiya R. Mustafina, Maxim V. Berezovski

**Affiliations:** 1 Department of Chemistry and Biomolecular Sciences, University of Ottawa, Ottawa, ON K1N 6N5, Canada; gyaro018@uottawa.ca (Y.A.G.); ezari068@uottawa.ca (E.A.Z.); 2 A.E. Arbuzov Institute of Organic and Physical Chemistry, Kazan Scientific Center, Russian Academy of Sciences, 420111 Kazan, Russia; gsl@analit-spb.ru (S.L.G.); svetlana@iopc.ru (S.V.F.); asiyamust@mail.ru (A.R.M.)

**Keywords:** DNA aptamers, silica nanoparticles, leukemia cells, luminescence, probes

## Abstract

DNA aptamers have many benefits for cell imaging, such as high affinity and specificity, easiness of chemical functionalization, and low cost of production. Among known aptamers, Sgc8-aptamer was selected against acute lymphoblastic leukemia cells with a dissociation constant in a nanomolar range. The aptamer was previously used for the covalent coupling with fluorescent and magnetic nanoparticles, as well as for the fabrication of aptamer-based biosensors. Among commonly used fluorescent tags, lanthanide nanoparticles offer stable luminescence with narrow, well-resolved emission peaks and the absence of photoblinking. In other words, lanthanide nanoparticles could serve as luminescence reporters and be used in biosensing. In our study, we conjugated amino- and carboxyl-modified silica-coated terbium (III) thiacalix[4]arenesulfonate luminescent nanoparticles with Sgc8-aptamer and showed the ability of the aptamer-conjugated nanoparticles to detect leukemia cells using fluorescence microscopy. In addition, we conducted a cell viability assay and confirmed that the nanoparticles do not induce spontaneous cell apoptosis or necrosis and could be potentially used for bioimaging applications.

## 1. Introduction

Among various types of cancers, leukemia is still recognized as one of the most common lethal cancers, even though a lot of advancements have been made for its diagnosis at early stages as well as for efficient therapy [1]. One of the major reasons that make leukemia hard to diagnose or cure is the fact that all abnormal white blood cells are surrounded by healthy blood cells, and it is quite challenging to distinguish between them. During standard chemotherapy, normal white blood cells are often damaged, which causes the downregulation of the immune system. To solve this problem, molecular agents specific to leukemia cells should be developed and applied for cancer cures.

Nucleic acid-based aptamers are cell-specific agents. They are short single-stranded DNA or RNA sequences selected through a process called systematic evolution of ligands by exponential enrichment (SELEX) [2]. Aptamers show strong and specific binding to cancer targets [3,4], low immunogenicity, low batch-to-batch variability, and are inexpensive [5]. They can be conjugated with drugs for targeted drug delivery [6,7], with fluorescent nanoparticles for cell imaging [8], and with magnetic nanoparticles for magnetic hyperthermia [9].

Tan and his colleagues pioneered aptamer selection against leukemia cells and presented a panel of selected oligonucleotides for specific recognition of human acute lymphoblastic leukemia cells [10]. Among the aptamers, Sgc8-aptamer has the highest affinity against malignant T cells with the lowest dissociation constant of 0.80 ± 0.09 nM and has been employed in many studies for efficient detection of leukemia cells using nanoparticles or aptamer-based biosensors [11,12,13,14,15]. In this article, we utilized Sgc8-aptamer for leukemia cell detection and conjugated it with lanthanide-based nanoparticles to create functional nanomaterials.

Lanthanide-based nanoparticles have gained a great attraction in biomedical research because they possess large Stokes shifts and sharp emission peaks in the UV, visible, and near-infrared (NIR) regions depending on the lanthanide ion [16,17,18,19]. In addition, lanthanide ions show long luminescence lifetimes and small photobleaching [20]. Calix[n]arenes coordinate lanthanide ions through covalently attached functional groups acting as antennae, enhancing both the luminescence and magnetic relaxation properties of lanthanides [21]. Indeed, terbium (III) complexes with thiacalix[4]arenesulfonate (TCAS) have the most intensive luminescence among other lanthanide-sulfocalixarene complexes [22]. TCAS coordinates Tb(III) through sulfonate groups of the upper rim in acidic/weakly acidic media and by phenolate groups of the lower rim in neutral/weakly basic media [23].

In this study, we conjugated Sgc8-aptamer with luminescent [Tb(TCAS)]-doped silica nanoparticles (SNs) via immobilized carboxyl and amino groups using the Michael addition-Shiff base reaction. The resulted aptamer-modified nanoparticles were applied for the detection and imaging of leukemia cells. It creates an opportunity for the lanthanide-based nanoparticles to be further applied in clinical diagnostics of leukemia.

## 2. Materials and Methods

### 2.1. Reagents and Materials

Tetraethyl orthosilicate (TEOS, 98%), ammonium hydroxide (28–30% in water), *n*-heptanol (98%), 3-aminopropyltriethoxysilane (99%), succinic anhydride (99%), *n*,*n*-dimethylformamide (DMF, 99.5%), β-alanine, fluorescamine, and acetic acid were purchased from Acros Organics (Oakville, Canada) and used without further purification. Terbium(III) nitrate hexahydrate (99.9%), *n*-hydroxysulfosuccinimide (Sulfo-NHS), and glutaraldehyde (50% wt in H_2_O) were from Alfa Aesar (Tewksbury, USA). Triton X-100, cyclohexane (99%), NaH_2_PO_4_, Na_2_HPO_4_, NaCl, Dulbecco’s phosphate-buffered saline (PBS, 1× with Ca/Mg), Bovine serum albumin (BSA), and 4-morpholineethanesulfonic acid hydrate (MES, 99%) were purchased from Sigma–Aldrich (Oakville, Canada). *n*-(3-dimethylaminopropyl)-*n*′-ethylcarbodiimide hydrochloride (EDAC) was purchased from Fluka Analytical (Buchs, Switzerland). Na_2_B_4_O_7_ was purchased from EMD Chemicals, Inc. (Philadelphia, PA, USA).

DMF, Ethanol, and TEOS were purified by distillation. The synthesis of *p*-sulfonatothiacalix[4]arene tetrasodium salt (TCAS) was carried out according to the procedure described in [23].

### 2.2. Oligonucleotides

Single-stranded DNA aptamers with a conjugated FAM dye—Sgc8-FAM (5′-*FAM*-TTT TTT TTT TAT CTA ACT GCT GCG CCG CCG GGA AAA TAC TGT ACG GTT AGA-3′) and a conjugated amino-group—Sgc-8-NH_2_ (5′-*NH_2_*-TTT TTT TTT TAT CTA ACT GCT GCG CCG CCG GGA AAA TAC TGT ACG GTT AGA-3′), the FAM-labeled N40 DNA library (5′-*FAM*-CT CCT CTG ACT GTA ACC ACG NNN NNN NNN NNN NNN NNN NNN NNN NNN NNN NNN NNN NNN NGG CTT CTG GAC TAC CTA TGC-3′) were purchased from IDT DNA Technologies (Coralville, IA, USA).

### 2.3. Cell Lines

CCRF-CEM (CCL-119, T lymphoblasts, acute lymphoblastic leukemia), Raji (CCL-86, Burkitt’s lymphoma, B lymphocytes), and Jurkat cell (TIB-152, Clone E6-1, T lymphocytes, acute T cell leukemia) lines were purchased from American Type Culture Collection (ATCC) and grown in RPMI media 1640 (1×, Gibco by Life Technologies, Burlington, Canada) supplemented with 10% FBS (Gibco by Life Technologies) and 1%–2% of antibiotics (Streptomycin-Penicillin, Gibco by Life Technologies, ref. 15140-122). Cells were maintained in the cell culturing incubator with a humidified atmosphere of 37 °C with 5% CO_2_.

### 2.4. Synthesis of Nanoparticles

Synthesis of amino-modified [Tb(TCAS)]-doped silica nanoparticles SNs-NH_2_ was performed according to the procedure published in [16,24]. Carboxyl-modified [Tb(TCAS)]-doped silica nanoparticles SNs-COOH were synthesized using the protocol published in [25]. The extent of the substitution of amino- to carboxyl-groups was measured using the fluorescamine-based procedure at pH 9 [26].

Bioconjugation of SNs-COOH by 5′-NH_2_-Sgc8 aptamer was performed according to the protocol [27] using EDAC and sulfo-NHS. Briefly, 1 mg of EDAC, 2.5 mg of sulfo-NHS, and 0.05 mL of 5′-NH_2_-Sgc8 aptamer (10 μM) were added to the dispersion of SNs-COOH (1 g/L, 1 mL) in MES (100 mM, pH = 5.65), and then incubated at room temperature with gentle shaking for 3 h. Afterwards, nanoparticles were washed three times with PBS (C = 100 mM, pH = 7.4) and dispersed in PBS + BSA (0.05%) solution for 1 h. Aptamer-conjugated nanoparticles Sgc8-SNs-COOH were washed with Na_2_B_4_O_7_ (0.05 M, pH = 9, with 1% BSA) and stored at 4 °C.

Synthesis of SNs-COH and their bioconjugation by 5′-NH_2_-Sgc8 aptamer Sgc8-SNs-COH were performed according to the protocol [27]. A dispersion of SNs-NH_2_ (1 g/L, 1 mL) was washed two times with PBS (100 mM, pH = 7.4, 1 mL). After the second wash, nanoparticles were suspended in 1 mL of 8% glutaraldehyde solution in PBS. The reaction was carried out for 6 h at room temperature with gentle shaking at 600 rpm. *SNs-COH* were washed two times with PBS (0.5 mL) and centrifuged. 1 mL of 5′-NH_2_-Sgc8 aptamer solution (10 μM in PBS) was added to the washed nanoparticles (pH = 7.4), and the nanoparticles were left at room temperature with shaking for 4 h. Afterwards, modified nanoparticles were washed two times with PBS, and the quenching solution was added (30 mM glycine + 0.05% BSA in PBS). After 30 min, *Sgc8-SNs-COH* nanoparticles were centrifuged again and suspended in the storage buffer (PBS + 0.05% BSA) and placed at 4 °C.

The quantitative analysis of amino groups on the surface silica nanoparticles modified by APTES was based on the reported protocol using fluorescamine [26]. Asparagine solutions in the concentration range of 6.25 × 10^−3^–1 × 10^−1^ mM in 50 mM of borate buffer (pH = 9.0) and 0.924 M of fluorescamine were used as a standard to make the calibration curve.

For the quantitative analysis of Sgc8-aptamer on the surface of Tb-doped silica nanoparticles, Sgc8-aptamer solutions in the concentration range of 375 nM–1.5 µM in 50 mM of borate buffer (pH = 9.0) were used. All samples were dispersed in 50 mM of borate buffer (pH = 9.0), and the concentration of nanoparticles in both cases was 0.05 g/L. Excitation of samples was performed at 390 nm, and emission was detected at 485 nm.

### 2.5. Methods

Nanoparticles size and morphology were studied using an FEI Tecani G2 spirit Transmission Electron Microscope with a LaB6 emitter. The images were acquired under an accelerating voltage of 120 kV. Samples were ultrasonicated in absolute ethanol for 10 min and were then applied on 200 mesh copper grids with continuous formvar support films.

Zeta potential (ξ-potential) was measured using a Zetasizer NanoZS (Malvern Instruments, Worcestershire, UK) instrument and Dispersion Technology Software (Nano Series, copyright 2008). All samples were diluted in bi-distilled water filtered through a 0.45 µm Millipore nylon membrane filter (Millipore-Q water purification system). Samples were ultrasonicated for 30 min using an ultrasonication bath prior to the measurements.

UV-Vis spectra were obtained with a Cary Eclipse (Agilent, Santa Clara, CA, USA) spectrophotometer. All samples were dispersed in Millipore-Q water. Tb-TCAS-COOH and Tb-TCAS-NH_2_ concentration was 0.5 g/L, Sgc8 aptamer concentration was 10 µM.

Luminescence of amino- and carboxyl-modified [Tb(TCAS)]-doped nanoparticles was measured using a Cary Eclipse fluorescence spectrophotometer. All samples were prepared in bi-distilled water (Millipore-Q water purification system) with a concentration of Tb-TCAS nanoparticles of 0.05 g/L. Samples were excited at 330 nm.

Flow cytometric analysis was performed in order to evaluate Sgc8-aptamer binding to leukemia cells. Cells were centrifuged (200× *g*, 3 min, 4 °C) and washed with PBS two times. Then, cells were incubated at 4 °C with Sgc8 aptamer (200 nM), DNA library (200 nM), and Anti-PTK7 Antibody (5 µg/mL) for 30 min at the dark. After incubation, samples were washed three times in order to wash away all the unbound sequences and analyzed with a Gallios Flow Cytometer (Beckman Coulter, Brea, CA, USA). Propidium iodide was used for the detection of dead cells, and the only viable single-cell population was gated and analyzed.

Fluorescence microscopy images were acquired with a Nikon Ni-U ratiometric fluorescence microscope with dual excitation sources and an apo 60xw objective. Images were captured with an Orca R2 (Hamamatsu, Hamamatsu City, Japan) charge-coupled device camera and analyzed through ImagePro software (Media Cybernetics, Bethesda, MD, USA). Cells (6 × 10^6^ cells) were washed with 10 mL of PBS two times and incubated with Sgc8-SNs-COOH and Sgc8-SNs-COOH nanoparticles (5 µg/mL). After incubation, samples were washed two times with PBS and added dropwise to the polylysine-coated microscopy slides that allow better adhesion of suspension cells. After 10 min, the glass slides were washed with PBS two times and sealed for further analysis.

Cell viability assay with Annexin V-FITC and propidium iodide was performed according to the published protocol [28]. Briefly, 8 × 10^6^ CCRF-CEM cells were incubated with different concentrations (0 µg/mL, 25 µg/mL, 50 µg/mL, and 100 µg/mL) of Sgc8-SNs-COOH. After 24 and 48 h, cells were centrifuged and washed with 10 mL of PBS two times and suspended in 100 µL of the binding buffer. Then, 6 µL Annexin V-FITC/PI (Gibco by Life Technologies) mixture was added to all samples for 15 min at 4 °C in the dark. Finally, 400 µL of binding buffer was added to the samples, and they were analyzed with a Gallios Flow Cytometer (Beckman Coulter, Brea, CA, USA).

Statistical analysis was performed using the OriginPro software.

## 3. Results

### 3.1. Synthesis of silica nanoparticles and Their Modifications with Amino and Carboxyl Groups

**Figure 1:**
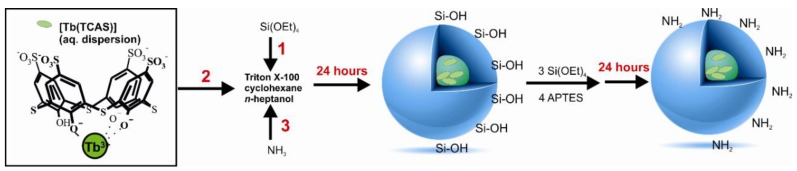
Synthesis of [Tb(TCAS)]-doped silica nanoparticles and their modification with amino groups (SNs-NH_2_). 1, 2, 3 are the order of adding reagents.

[Tb(TCAS)]-silica doped nanoparticles with immobilized amino or carboxyl groups were used and were further conjugated with Sgc8-aptamer. Amino-functionalized [Tb(TCAS)]-doped silica nanoparticles (SNs-NH_2_) were synthesized through the water-in-oil reversed microemulsion method commonly used for nanoparticles with core-shell structures [27]. Briefly, nanoparticles (NPs) were made using basic hydrolysis of tetraethylorthosilane (TEOS) in the presence of [Tb(TCAS)], then (3-aminopropyl)thriethoxysilane (APTES) was used in order to immobilize amino groups on the surface of silica nanoparticles according to the published protocol (Figure 1) [27].

As it was revealed with TEM, SNs-NH_2_ had a quasi-spherical shape and diameter of 39 ± 12 nm (Figure S1b). The number of immobilized amino groups per one NP with a diameter of 39 nm was approximately 4290 amino groups. The number of amino groups was determined using the fluorescamine method (Figure S2) [26]. [Tb(TCAS)]-doped silica nanoparticles with carboxyl groups (SNs-COOH) with a diameter of 39 nm were synthesized through the treatment of SNs-NH_2_ with succinic anhydride (Figure S1a). The amount of immobilized carboxyl groups per one NP was 1720 (conversion rate, 40%, Figure S2).

### 3.2. Conjugation of the Amino-Modified Aptamer with SNs

**Figure 2:**
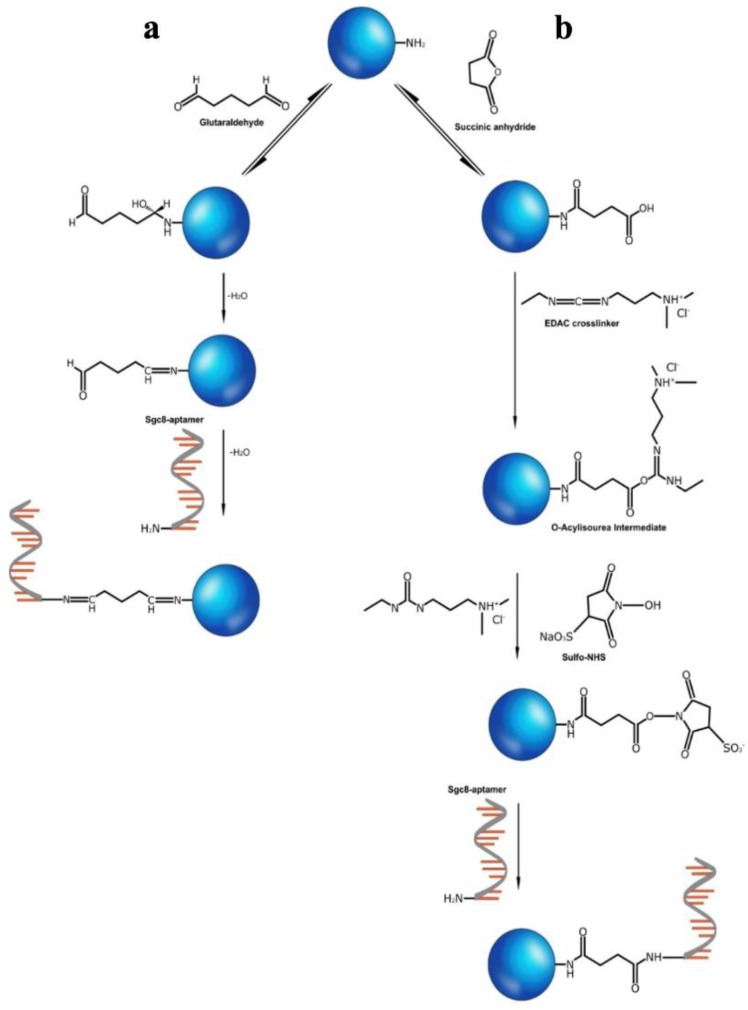
Conjugation of SNs-NH_2_ with Sgc8 aptamer using glutaraldehyde (**a**) or succinic anhydride and EDAC/Sulfo-NHS (**b**).

We used two approaches for the conjugation of SNs-NH_2_ with the amino-modified aptamer (Sgc8). The schemes of the reactions are shown in Figure 2. The first method implies SNs-NH_2_ treatment with glutaraldehyde in the Schiff base reaction with further cross-linking with Sgc8-aptamer [29]. In the second method, SNs-NH_2_ were transformed to SNs-COOH using succinic anhydride and treated with *n*-(3-dimethylaminopropyl)-*n*′-ethylcarbodiimide hydrochloride (EDAC) in the presence of *n*-hydroxysulfosuccinimide (Sulfo-NHS) to obtain amine-reactive Sulfo-NHS ester SNs for further immobilization of Sgc8-aptamer on the nanoparticles according to the published protocol [27].

**Figure 3:**
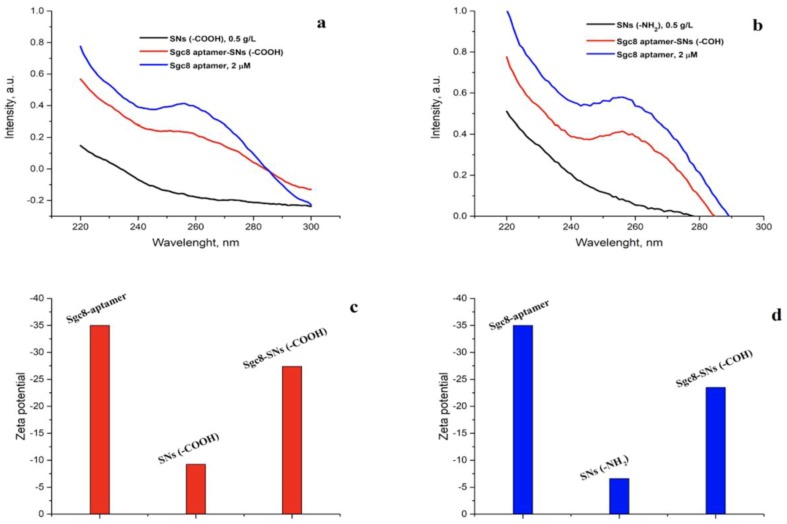
UV-absorbance spectra of Sgc8-aptamer (2 μM), SNs-COOH (C = 0.5 g/L), and Sgc8-SNs-(COOH) (C = 0.5 g/L) (**a**) and SNs-NH_2_ (C = 0.5 g/L) and Sgc8-SNs-(COH) (C = 0.5 g/L) (**b**) in water. Zeta potential values of Sgc8-aptamer, SNs-COOH, and Sgc8-SNs-(COOH) (**c**) and Sgc8-aptamer, SNs-NH_2_, and Sgc8-SNs-(COH) (**d**) measured in water.

To check for the presence of the aptamer on SNs, UV-absorbance spectra of the nanoparticles before and after conjugation were measured (Figure 3). It was found that a DNA absorbance peak at 260 nm was present in the samples after the cross-linking of Sgc8 with SNs using glutaraldehyde (Sgc8-SNs-COH) and succinic anhydride, EDAC/Sulfo-NHS reagents (Sgc8-SNs-COOH).

Figure 3c,d represents the zeta potential of Tb(III)-doped silica nanoparticles with immobilized amino and carboxyl groups. As expected, the zeta potential of Sgc8-aptamer was negative due to the phosphate backbones within the DNA structure, and SNs-NH_2_ zeta potential was higher than for SNs-COOH due to the partial negative charge on the carboxyl groups. It should be noted that the small difference in the zeta potential between SNs-COOH nanoparticles and SNs-NH_2_ nanoparticles could be due to the fact that some amino groups have not been substituted completely during the synthetic procedure. Thus, it increased the zeta potential value. However, successful conjugation can be explained by the decrease of the zeta potential for the Sgc8-SNs-COOH and Sgc8-SNs-COH samples due to the phosphate backbones contribution. Zeta potential values, as well as the concentration of all samples, are presented in Table S1.

Qualitative analysis of covalently bound Sgc8-aptamer was performed utilizing the fluorescamine method (Figure S3) [26]. For the calibration curve, we used aqueous solutions of Sgc8-aptamer in the concentration range of 37.5 nM–1.5 μM at pH = 9 (Figure S3a,b). As some of the primary amino groups remained unreacted (~2575 in the case of SNs-COOH, Figure S2), and could also react with fluorescamine, the amount of the conjugated Sgc8-aptamer was determined as the difference between luminescence spectra and was 0.66 μM for Sgc8-SNs-COH and 0.55 μM for Sgc8-SNs-COOH.

**Figure 4:**
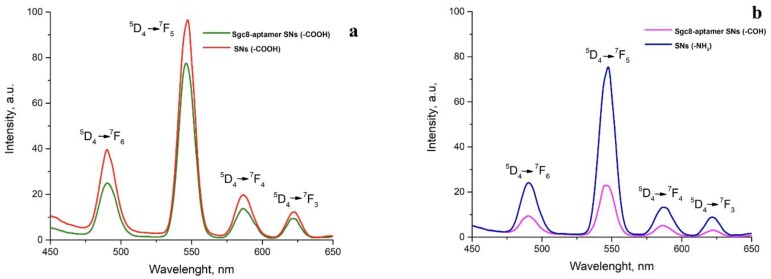
Emission spectra of SNs-COOH (**a**) and SNs-NH_2_ (**b**) before and after conjugation with Sgc8-aptamer. All spectra were obtained in water media under excitation wavelength—330 nm; concentration of nanoparticles—0.5 g/L.

Indeed, to show that the luminescence properties of nanoparticles after modification have not been affected, we obtained luminescence spectra for all samples. Figure 4 represents the luminescence spectra of amino- and carboxyl-modified silica nanoparticles before and after conjugation with Sgc8-aptamer. SNs-NH_2_, SNs-COOH, Sgc8-SNs-COOH, and Sgc8-SNs-COH possess four emission peaks, which are typical for Tb(III)-centered luminescence at 489 nm (^5^D_4_→^7^F_6_), 541 nm (^5^D_4_→^7^F_5_), 582 nm (^5^D_4_→^7^F_4_), and 620 nm (^5^D_4_→^7^F_3_). Interestingly, the luminescence of SNs-COOH and Sgc8-SNs-COOH is more intense compared with SNs-COH and Sgc8-SNs-COH at the same concentration (Figure 4).

The luminescence intensity of Sgc8-SNs-COOH at 550 nm was slightly decreased, although we did not observe any other significant changes in luminescence properties after nanoparticle modification. Nevertheless, SNs-NH_2_ showed the decreased luminescence intensity that could also be due to the interaction between amino groups and a silica shell that caused the aggregation of nanoparticles.

### 3.3. Detection of Leukemia Cells with Flow Cytometry and Fluorescent Microscopy

**Figure 5:**
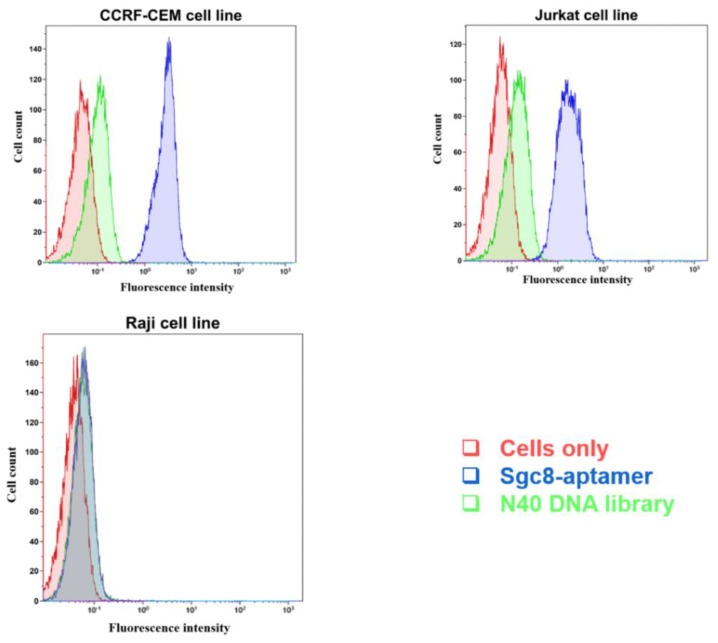
Flow cytometry analysis of the binding of the FAM-labeled Sgc8-aptamer with CCRF-CEM and Jurkat cells (PTK7 positive cells), and Raji cells (PTK7 negative cells). The N40 DNA library was used as a control for non-specific DNA binding.

Prior to the conjugation of SNs-NH_2_ and SNs-COOH with Sgc8-aptamer, we decided to confirm Sgc8-aptamer binding with CCRF-CEM, Jurkat, and Raji cell lines (Figure 5). Sgc8-aptamer showed strong binding with CCRF-CEM as well as the Jurkat cell line, although the shift of fluorescence intensity was stronger for CCRF-CEM cells than for Jurkat cells; Jurkat cells could also be used as a model for all further experiments. It was reported that Sgc8-aptamer binds to a tyrosine protein kinase 7 (PTK7). PTK7 has the other name of colon carcinoma kinase 4 (CCK4) and is involved in the transduction of extracellular signals across the cellular membrane [30]. A stronger shift of fluorescence intensity for CCRF-CEM could be associated with the higher amount of PTK7 on the cellular membrane than for Jurkat cells; however, both CCRF-CEM and Jurkat cell lines are PTK7-positive, as previously shown in other experiments with the anti-human PTK7 antibody [30].

**Figure 6:**
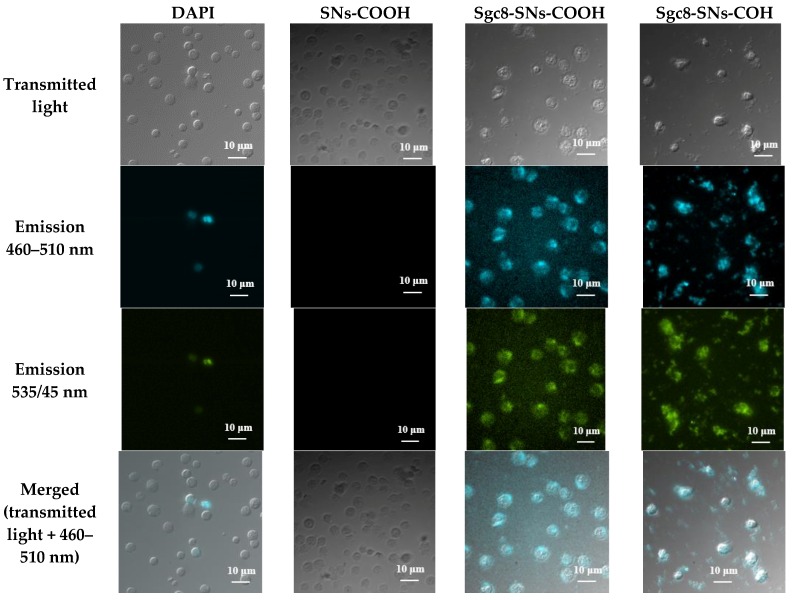
Fluorescent microscopy images of CCRF-CEM cells treated with DAPI, SNs-COOH (a control sample), Sgc8-SNs(COOH), and Sgc8-SNs(COH). Excitation wavelength—340 nm.

**Figure 7:**
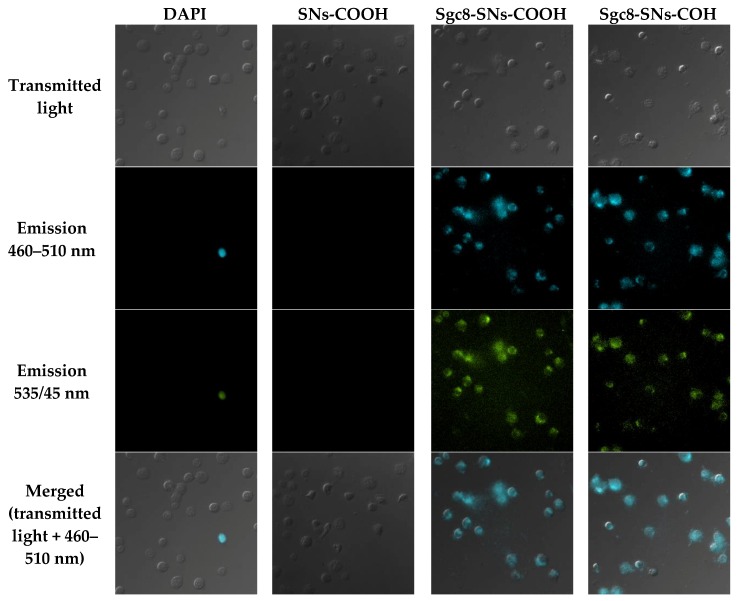
Fluorescent microscopy images of Jurkat cells treated with DAPI, SNs-COOH nanoparticles (a control sample), Sgc8-SNs(COOH), and Sgc8-SNs(COH). Excitation wavelength—340 nm. Scale bar: 10 μm.

Figure 6 and Figure 7 show fluorescence microscopy images of CCRF-CEM and Jurkat cells treated with SNs-COOH (a control), Sgc8-SNs-COOH, Sgc8-SNs-COH, and 4′,6-diamidino-2-phenylindole (DAPI). As expected, Sgc8-SNs-COOH and Sgc8-SNs-COH labelled CCRF-CCEM and Jurkat cells; however, no signal was obtained for the Raji cell line, which is known to have no PTK7 at the cell surface. Considering emission spectral overlap between DAPI and Tb(III) fluorescence, it is quite challenging to use them simultaneously within one sample; however, considering their broad emission spectrum and photostability, that creates promising possibilities for these nanoparticles to be used in bioimaging applications. Moreover, we decided to use non-fixed cells as this could show the difference between DAPI staining (it shows cells with the damaged membrane) and all cells targeted by Sgc8-SNs-COOH.

Interestingly, while DAPI stained only cells with a damaged cellular membrane (presumably, necrotic cells), SNs-NH_2_ and SNs-COOH conjugated with Sgc8-aptamer labeled all cells. Moreover, due to the wide emission spectrum of Tb-TCAS-doped SNs with maximum intensity peaks at 489 nm and 541 nm, they were observed in two fluorescence channels.

### 3.4. Cell Viability Assay

For in vivo experiments as well as clinical applications, it is essential to evaluate aptamer-conjugated nanoparticle cytotoxicity and the possible side effects that they may cause in living organisms. It has been reported previously that silica itself and silica-coated materials may induce some cytotoxic effects and should be carefully evaluated prior to being widely used as a matrix for targeted drug delivery and other biological applications [31]. In addition, lanthanide nanoparticles containing rare earth elements could be potentially cytotoxic. At the same time, there are no sufficient data published regarding the Tb compound’s cytotoxicity, even though it has incredible optical properties that could be employed in biological applications [32].

A number of experiments with yttrium group fluorides on animals and peritoneal macrophages (rat) were conducted in 1994 [33]. The authors mentioned that it was crucial to control the level of yttrium, terbium, and lutetium fluorides in the air of the workplace, with a maximum admissible concentration of fluorides of 2.5 mg/m^3^ (the maximum individual concentration). Later, in 1996, pulmonary toxicity of systemic Tb was examined using mice [34]. It was concluded that intravenous administration of Tb caused pulmonary lipid peroxidation at early stages, and also that SOD, CAT, and GSH-Px could be acting as possible modulators of lipid peroxidation induced by Tb ions. On the other hand, effects of lanthanum, cerium, yttrium, and terbium ions on the respiratory burst of peritoneal macrophages were studied, and it was stated that lanthanide ions could inhibit the production of active oxygen species at high concentrations [35].

We checked cell viability with Annexin V-FITC and PI in order to determine the percentage of viable, apoptotic, necrotic, and viable cells with a damaged cell membrane as described elsewhere [28]. Annexin V acts as a ligand to phosphatidylserines that are overexpressed on the cellular membrane of apoptotic cells, while PI is used for staining all cells with a damaged cellular membrane as it can easily penetrate a membrane and bind to double-stranded DNA in the same way as DAPI and 7-AAD. Thus, cells stained with both PI and Annexin V-FITC are necrotic.

CCRF-CEM cells were treated with various concentrations (0 µg/mL, 25 µg/mL, 50 µg/mL, and 100 µg/mL) of Sgc8-SNs-COOH for 24 and 48 h. Afterwards, cells were centrifuged and washed with PBS and stained with Annexin V-FITC and PI for further analysis with flow cytometry.

**Figure 8:**
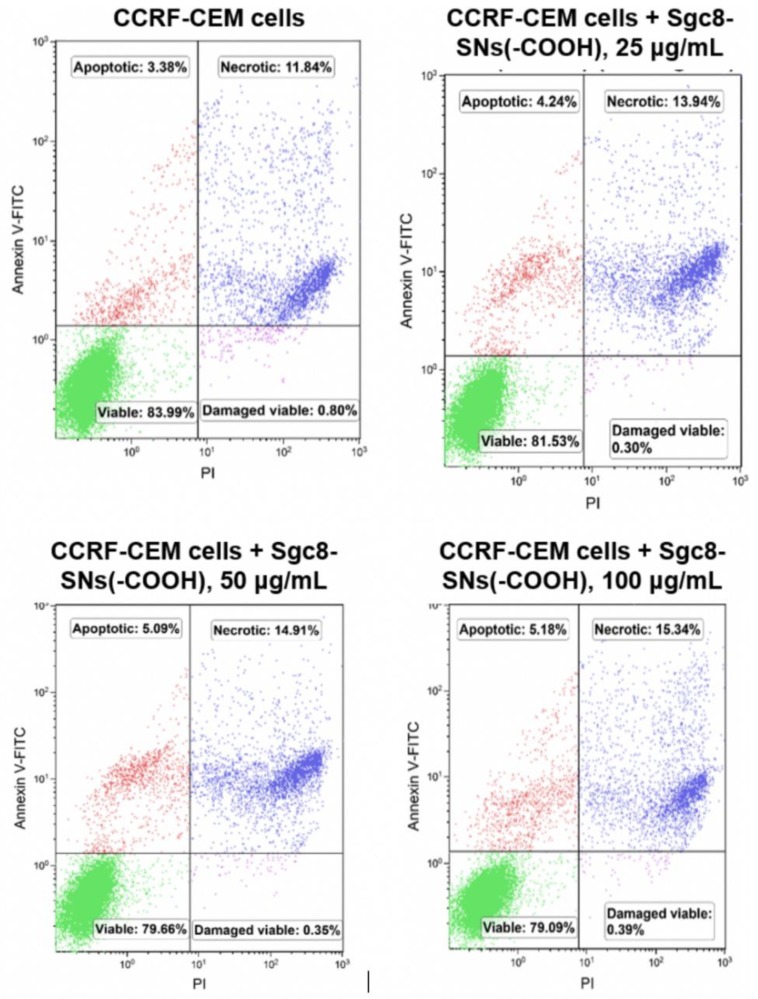
Cell viability assay with Annexin V-FITC and PI after 24 h of incubation. Sgc8-SNs(COOH) concentration: 0 µg/mL (**a**), 25 µg/mL (**b**), 50 µg/mL (**c**), and 100 µg/mL (**d**).

Figure 8 shows that the amount of CCRF-CEM cells that underwent apoptosis and necrosis has slightly increased after 24 h incubation with Sgc8-SNs-COOH comparing to the control sample without NPs. Indeed, the increase of NP concentration from 25 µg/mL to 100 µg/mL had a modest effect on the cells’ viability. A similar pattern was observed after 48 h of incubation (Figure S4), with roughly 5% of the cells turning necrotic, which proves that Sgc8-SNs-COOH do not induce significant toxic effects and do not cause spontaneous apoptosis or necrosis of the cells.

## 4. Conclusions

In this study, we presented an efficient and facile way for conjugation of Tb-TCAS-doped silica nanoparticles with immobilized carboxyl and amino groups with Sgc8-aptamer using the Michael addition-Shiff base reaction. In a simple manner, the synthetic procedure allows the conjugation of biomolecules with functional nanomaterials in three steps. It should be noted that nanoparticles retained their luminescent properties, although SNs-NH_2_ coupled with Sgc8-aptamer possessed less intensive luminescence to compare to free nanoparticles.

Sgc8-aptamer was chosen for modification with SNs because it showed a high affinity to a cancer-related target, PTK7, with the binding constant in the nanomolar range. We confirmed the ability of Sgc8 to bind CCRF-CEM and Jurkat cells with flow cytometry selectively, but not with the Raji, a PTK7-negative cell line. We showed that Sgc8-SNs-COOH and Sgc8-SNs-COH could selectively detect positive cell lines using fluorescent microscopy. This creates an opportunity for our modified nanoparticles to be employed for bioimaging and biosensing.

Moreover, we assessed the cytotoxicity of Sgc8-SNs-COOH nanoparticles using cell viability assay with Annexin V-FITC and PI and revealed that the nanoparticles did not induce apoptosis or necrosis of leukemia cells. The results make Sgc8-SNs-COOH nanoparticles a promising agent for diagnosis and therapy of acute myeloid leukemia.

## Supplementary Materials

Supplementary materials can be found at https://www.mdpi.com/2227-9059/8/1/14/s1.
