# Supplementary material for: Aptamer-Conjugated Tb(III)-Doped Silica Nanoparticles for Luminescent Detection of Leukemia Cells
Source: Biomedicines. 2020 Jan 13;8(1):14. doi: 10.3390/biomedicines8010014 (PMC7168109; doi:10.3390/biomedicines8010014)
Supplement: Supplementary file 1 [file biomedicines-08-00014-s001.pdf]

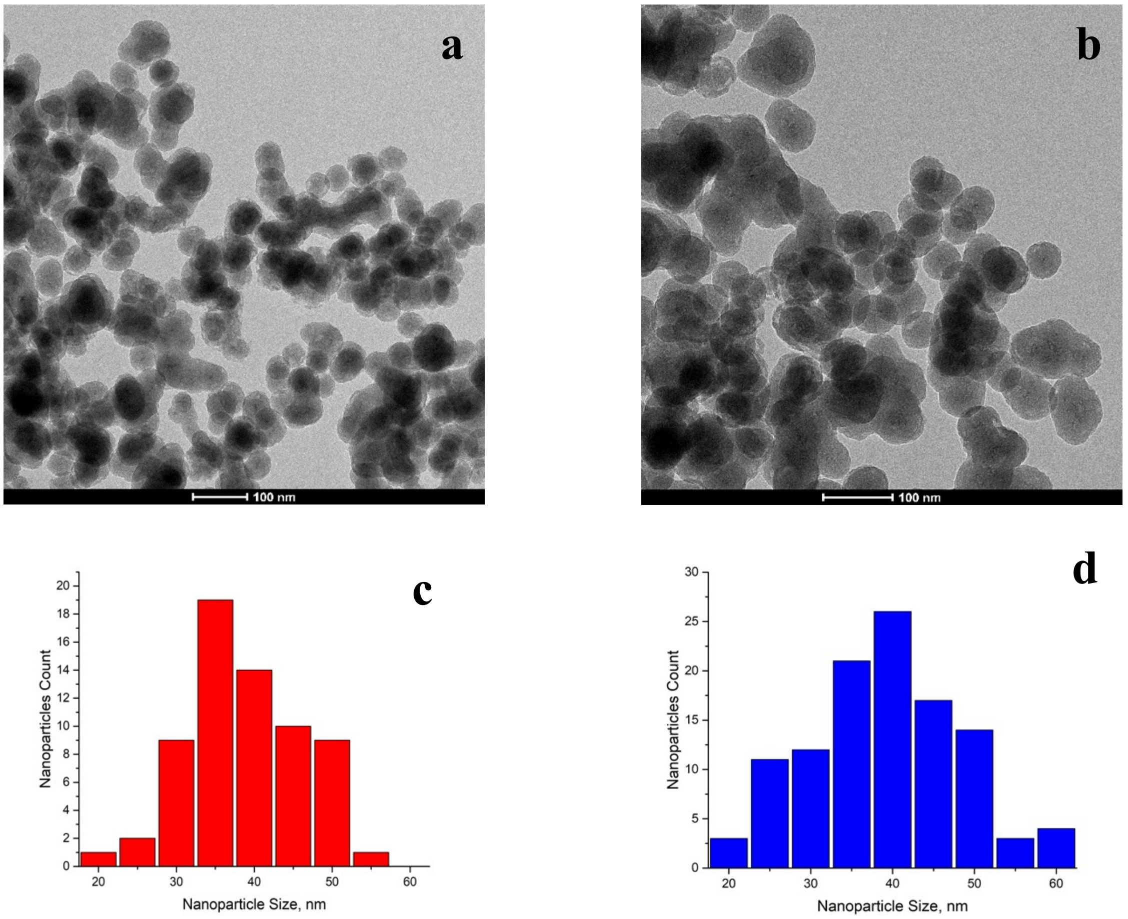

Figure S1. TEM images of SNs-COOH and SNs-NH_2 (a, b) and nanoparticles size distribution (c, d). Average diameter of SNs-COOH – 39±10 nm, SNs-NH_2 – 39±12 nm.

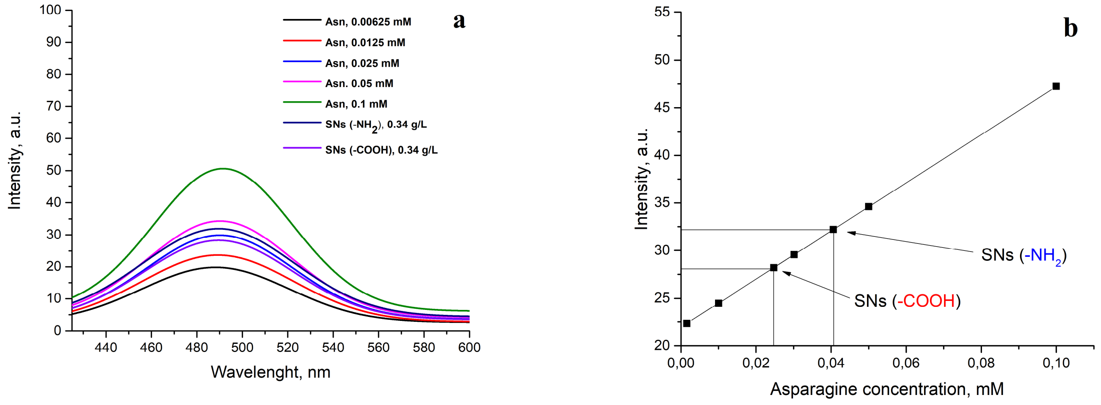

Figure S2. (a) Fluorescamine emission spectra with various concentration of asparagine (0.00625 mM, 0.0125 mM, 0.025 mM, 0.05 mM, 0.1 mM) and SNs-NH2 (0.34 g L^{-1}) and SNs-COOH (0.34 g $^{L-1}$). All spectra were obtained in 50 mM borate buffer, [Fluorescamine] = 0.924 M under excitation wavelength – 390 nm. (b) Calibration curve.

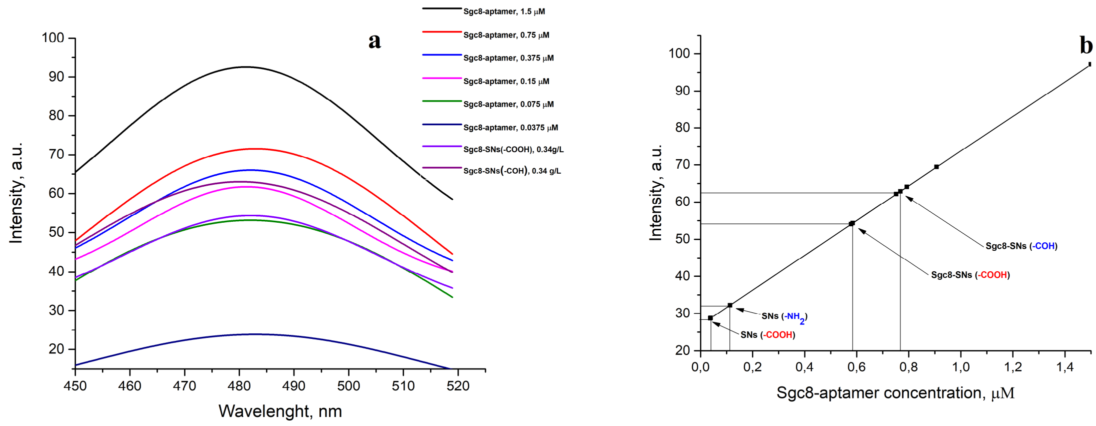

Figure S3. (a) Fluorescamine fluorescence spectra after interaction with various concentration of Sgc8-aptamer (1.5 μM, 0.75 μM, 0.375 μM, 0.15 μM, 0.075 μM, 0.0375 μM) and SNs-NH2, SNs-COOH, Sgc8-SNs(COH) (0.34 g $^{L-1}$) and Sgc8-SNs(COOH) (0.34 g $^{L-1}$). All spectra were obtained in 50 mM borate buffer under excitation wavelength – 390 nm. (b) Calibration curve.

Table S1. Zeta potential values and concentration of Sgc8-aptamer, SNs-COOH, SNs-NH_2, Sgc8-SNs (-COOH) and Sgc8-SNs (-COH) in water.

Sample	Concentration of NPs, g L^{-1}	Concentration of aptamers, μM	ξ-Potential, mV
Sgc-8-NH_2 aptamer	-	0.1	-35
	-	0.5	-29
SNs-NH_2	0.01	-	-7
	0.05	-	-4
Sgc8-SNs(COH)	0.01	-	-24
	0.05	-	-22
SNs-COOH	0.01	-	-9
	0.05	-	-9
Sgc8-SNs(COOH)	0.01	-	-27
	0.05	-	-23

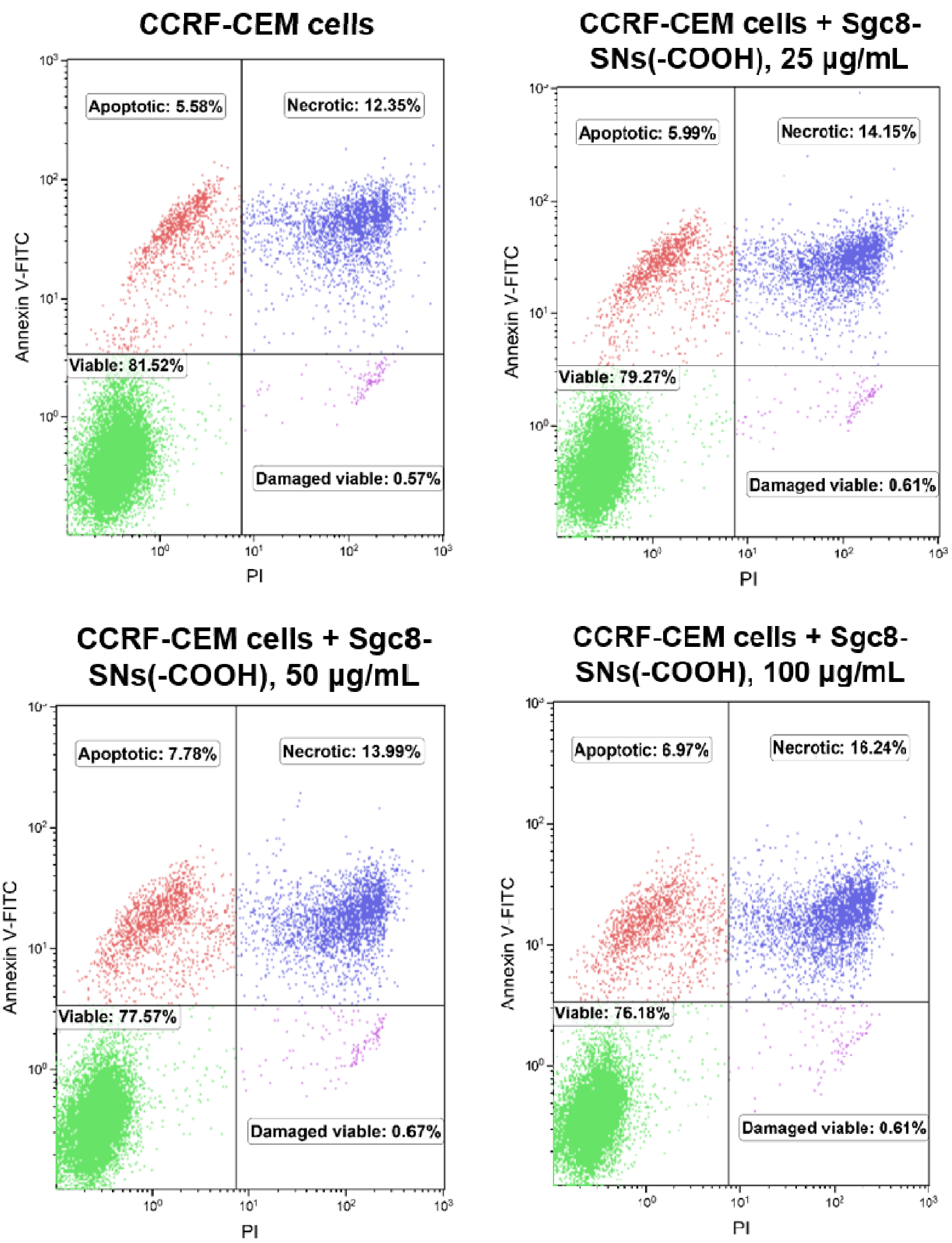

Figure S4. Cell viability assay with Annexin V-FITC and PI after 48 hours of incubation. Sgc8-SNs(COOH) concentration: 0 μg/mL (a), 25 μg/mL (b), 50 μg/mL (c) and 100 μg/mL (d).
